# Scope of FNAC in the diagnosis of soft tissue tumors-A study from a tertiary cancer referral center in India

**DOI:** 10.1186/1742-6413-4-20

**Published:** 2007-10-31

**Authors:** Bharat Rekhi, Biru D Gorad, Anagha C Kakade, RF Chinoy

**Affiliations:** 1 Dept of Pathology, Tata Memorial Centre, Dr E. B. Road, Parel, Mumbai, 400012, India; 2 Clinical Trials Coordinator, Epidemiology & Clinical Trials Unit (ECTU), Advanced Centre for Treatment, Research & Education in cancer (ACTREC), Tata Memorial Centre, Khargar, Navi Mumbai, India

## Abstract

**Background:**

Fine needle aspiration cytology (FNAC) forms one of the first diagnostic tools in the evaluation of tumors. Its role in diagnosing soft tissue tumors (STT) has been fairly documented, as well as debated. Present study was aimed at evaluating its scope in diagnosing 127 cases of soft tissue tumors.

**Methods:**

Conventional Pap and MGG staining was available in all the cases. Immunocytochemistry (ICC) was performed in 15 cases. Histopathological details were available in 115 cases.

**Results:**

50% cases were referred for a primary diagnosis, while 26.8% & 22.8% cases were evaluated for recurrent and metastatic lesions, respectively. Extremities were the commonest sites. On FNAC, 101 cases (79.5%) were labeled as malignant, whereas 10 cases (7.9%) were labeled as benign. The remaining 16 cases (11%) were not categorized and were labeled as 'unsure/not specified'. Histopathological confirmation in 115 cases, gave a diagnostic accuracy of 98%, with a positive predictive value of 98% in malignant cases and a negative predictive value of 100% in benign cases. Two cases were false positive. Among the various cytological categories, 60 cases (47.2%) were of spindle cell type, followed by 32 (25.2%) of round cell type and 14 cases (11%) of lipomatous type. Other 12 cases (9.4%) were of pleomorphic type; 7 (5.5%) cases of epithelioid type and remaining 2 cases were of myxoid type. All the round cell, pleomorphic and myxoid type of tumors were sarcomas, whereas 73.3% cases of spindle cell type were labeled as 'malignant'. Exact cytological sub typing was offered in 58 cases, with rhabdomyosarcoma (RMS) as the most frequently sub typed tumor. The two false positive malignant cases were of fibromatosis and a pigmented schwannoma, on biopsy. Out of 28 metastatic lesions, lymph nodes were the commonest site for metastasis, with epithelioid tumors that formed highest percentage of metastatic cases.

**Conclusion:**

FNAC is fairly specific and sensitive in STT diagnoses for primary, recurrent and metastatic lesions. The cytological types, especially round cell and pleomorphic sarcomas, can be quickly identified. Clinicopathological correlation with ICC as an adjunct, are valuable in exact sub typing.

## Background

Soft tissue tumors (STT) are rare neoplasms. At our hospital, 2.5% of the cancer cases are constituted by soft tissue sarcomas. Most of these cases are referrals, including a larger proportion of malignant cases, invariably with a high grade and stage. [1] These tumors pose a significant diagnostic challenge as a result of their morphologic overlap and biological heterogeneity [2]. Among its various diagnostic aids, lately, FNAC has been gaining importance as a result of its cheap cost, easy performance, safety, along with fair specificity and specificity, especially in terms of sorting out malignant cases. Apart from its use in recurrent and metastatic cases, FNAC has been identified as a useful diagnostic technique in the initial diagnosis of STTs [3-8].

The present study was aimed at evaluating 127 cases of FNAC smears from soft tissue tumors, diagnosed over a period of 5 years. The other objectives included identification of cyto-histological concordance in terms of malignancy; stratification of the tumors into various cytomorphological categories and to note the extent of specific sub-typing in individual cases.

## Methods

127 aspirates (unaided and guided) from STTs were evaluated over a period of 5 years (2001–2006). Only cases with relevant clinical data were included. All cases included conventional Papanicolaou (Pap) and May Grunwald Giemsa (MGG) stained smears. Inadequate smears were the ones with 'no' or scanty cells. Non-neoplastic lesions, on biopsy, were excluded. Histological details, in 115 cases, were accessed from the clinical charts and the Hospital Diagnostic Information System (DIS).

On examination, all cases were placed into 6 cytomorphological categories, namely spindle cell, round cell, pleomorphic, myxoid, epithelioid/polygonal cell and lipomatous type [9]. In cases of mixed components, the specific subtype was assigned based on the predominant morphological pattern. Immunocytochemistry was performed in 15 cases for an exact sub typing, on smears and cell blocks. Exact sub typing was offered in the clinical context, with ICC as an adjunct.

### Immunocytochemistry (ICC) technique on smears

Smears were fixed in methanol, followed by 3 changes in 100% absolute alcohol for 5 min each. This was followed by washing under running tap water for 5 min. Next, the smears were subjected to endogenous peroxidase for 30 min (H_2_O_2 _+ methanol). This was followed by washing under running tap water, antigen retrieval (low heat with microwave or enzyme) and 3 changes of wash with Tris Buffer solution (TBS) ~5 min. Subsequently, blocking serum was applied, followed by incubation with primary antibody ~1 hour and TBS washing. Further, secondary antibody was applied for 30 min, followed by washing with TBS and application of avidin-biotin complex (ABC). Afterwards, TBS washing was carried out with subsequent staining with Diamino Benzidine (DAB) reagent. Harris' hematoxylin was used as a counter stain. Finally, the smears were dehydrated with absolute alcohol, cleared with xylene and mounted with DPX. The cases were accompanied with appropriate controls. A limited panel of markers was carried out on the smears, including vimentin, desmin, S-100, cytokeratin (CK), MIC-2, myogenin, neuron specific enolase (NSE), synaptophysin, chromogranin and myeloperoxidase (MPO).

Immunohistochemistry (IHC) was performed by immunoperoxidase procedure. On biopsy a wide panel of markers was carried out for exact subtyping. In addition, smooth muscle actin (SMA), Myo D-1, BCL-2, epithelial membrane antigen (EMA), Leukocyte common antigen (LCA), *ALK*-1, CD30, NSE, synaptophysin and chromogranin (Dako, Produkionsveg, Glostrup, Denmark) constituted as other markers.

### Statistical analysis

Statistical analysis was carried out using SPSS (version 14) software. Descriptive analysis was performed using frequency and percentages. In terms of malignancy, cytology was compared with histopathological findings by calculating specificity, sensitivity, and concordance along with positive and negative predictive values (PV). The significance of various cytological categories in terms of benign, malignant and 'unsure'/not specified' was analyzed using chi-square test. P value < 0.05 was considered significant.

## Results

Out of 127 cases, maximum cases (27, 21.3%) were noted in the age group 21–30 years. Lower extremities were the common sites, with thigh as the most common site of occurrence in 20 cases (15.7%). Males outnumbered the females (1.8:1).

Sixty-four cases (50.4%) were referred for a primary diagnosis, while 34 cases (26.8%) were evaluated for recurrent lesions & remaining 29 cases (22.8%) cases for metastatic disease.

On FNAC, 101 cases (79.5%) were labeled as malignant and 10 cases (7.9%) as benign. 16 cases (14.5%) were labeled as 'unsure'/'not categorized'. With biopsy results, finally, 107 cases (84.25%) were labeled malignant/sarcomas; 16 (12.59%) as benign, 2 as borderline/intermediate malignancies and 2 cases were 'unsure/not specified'. Histopathological confirmation, available in 115 cases (90.5%), gave a diagnostic accuracy of 98% with a positive predictive value of 98% in terms of malignancy and a negative predictive value of 100% in benign cases. The overall sensitivity was 100% and specificity was 83.3%.

Out of the 16 cases that were not categorized, 5 turned out to be benign, 8 malignant and 2 cases as intermediate malignancy, on histopathology. The remaining 2 cases lacked biopsy results.

Among the various cytomorphological categories, 14 cases (11%) were of lipomatous tumors, 12 cases (9.4%) were of pleomorphic type, 32 cases (25.2%) were of round cell type, 60 (47.2%) of spindle cell type, 7 cases (5.5%) of epithelioid type and the remaining 2 cases were of myxoid type. All the round cell, epithelioid, pleomorphic and myxoid tumors were sarcomas. Out of 60 cases of spindle cell subtype, 44 (73.3%) were malignant and 16 cases (26.6%) were not categorized, either as benign or malignant.

The cytological diagnoses included 'terms' like spindle cell sarcoma (31 cases), malignant round cell tumor (12 cases), sarcoma of epithelioid type (7 cases), spindle cell tumor (14 cases), and spindle cell lesion (4 cases). Ten benign cases were of lipomas.

Out of 16 'unsure'/'non classified' cases; on biopsy, 8 cases turned out to be malignant, namely 3 spindle cell sarcomas, 2 MPNSTs, 1 synovial sarcoma, 1 gastrointestinal stromal tumor (GIST) and 1 pleomorphic sarcoma (NOS). The remaining 8 cases included 2 of intermediate malignancy, namely, 1 case, each of a dermatofibrosarcoma protuberans (DFSP) and fibromatosis. Another 1 case was of proliferative fasciits. Another 3 cases included 2 of schwannoma and 1 case of a neurofibroma. Remaining 2 cases could not be categorized due to lack of biopsy results

With the help of clinical details and ICC, exact sub typing was possible in 58 cases. Out of 14 lipomatous tumors, commonest were lipomas (10 cases), including 1 case of a pleomorphic lipoma; 2 cases were of myxoid liposarcomas and remaining 2 cases were of pleomorphic liposarcomas. Out of 60 cases of spindle cell category, specific diagnoses in 14 cases included leiomyosarcoma (3 cases), MPNST (2 cases), synovial sarcoma (3 cases), melanoma of soft parts (1 case). One case was offered a differential diagnosis of MPNST vs. melanoma of soft parts, another of a MPNST vs. synovial sarcoma and remaining 1 case was of sarcoma vs. a sarcomatoid carcinoma (poorly differentiated). In the round cell category, exact sub typing was more possible i.e. in 20 cases that included RMS (10 cases), PNET/Ewing's (9 cases) along with 1 case of a neuroblastoma. In the pleomorphic group, 8 cases were labeled as pleomorphic cell sarcoma, 2 as pleomorphic rhabdomyosarcomas and 1 case as an extraskeletal osteosarcoma. One case was offered a differential of a pleomorphic sarcoma vs. poorly differentiated carcinoma, however, lacked biopsy results.

**Figure 1:**
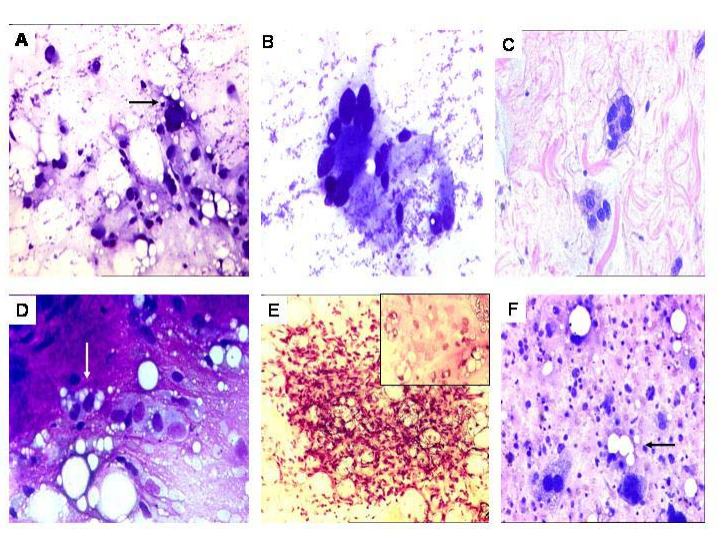
A. Pleomorphic lipoma. Mature adipose tissue fragments with giant cells (arrow). MGG × 200. B. High power view of 'Floret-type' giant cells, displaying 'wreath-like' nuclear arrangement. MGG × 400. C. Biopsy of pleomorphic lipoma displaying floret-type giant cells. H&E × 200. D. Myxoid Liposarcoma. Abundant metachromatic, myxoid stroma with vacuolated cells (Lipoblasts) (arrow). MGG × 400. E. Cellular smear showing plexiform capillary network with entrapped lipoblasts (inset) Pap × 200. F. Pleomorphic liposarcoma. Hypercellular smear showing several pleomorphic cells (arrow), including lipoblasts admixed with inflammatory cells. Pap × 400.

**Figure 2:**
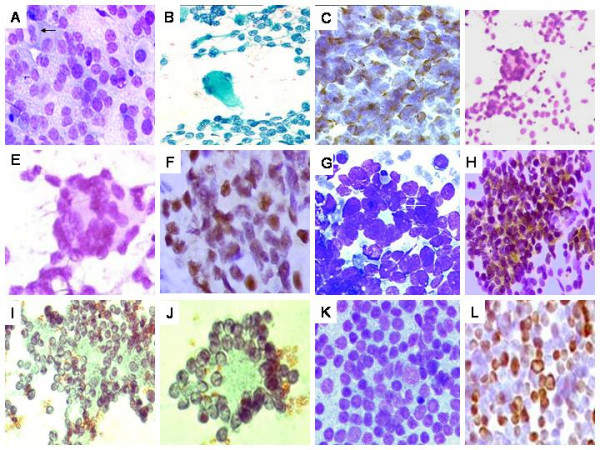
A. Rhabdomyosarcoma (RMS). Hypercellular smear with round cells, including plasmacytoid and binucleate forms (arrow), against a 'lacy' background MGG × 200. B. Smear with singly scattered round cells and an isolated strap cell/tumor rhabdomyoblast (arrow). Pap × 200. C. Diffuse desmin positivity in the round cells. DAB × 400. D. Alveolar RMS. Hypercellular smear with round cells including 'wreath'-like giant cells (inset). Pap × 400. E. High power view of an isolated wreath-like giant cell. Pap × 400. F. Cell block preparation showing strong myogenin expression. DAB × 400. G. PNET. Hypercellular smear with round cells, scattered singly and forming 'rosettes' (arrow). H. Positive MIC2 (CD99) expression in the tumor cells (DAB × 400). I. Neuroblastoma. Smear displaying round cells, with fine nuclear chromatin and exhibiting Homer-wright rosettes. J. High power view of an isolated pseudo rosette. L. Granulocytic sarcoma (formed on biopsy). Malignant round cell tumor, showing blasts. Strong MP0 positivity, on imprint, in the tumor cells. (DAB × 200).

**Figure 3:**
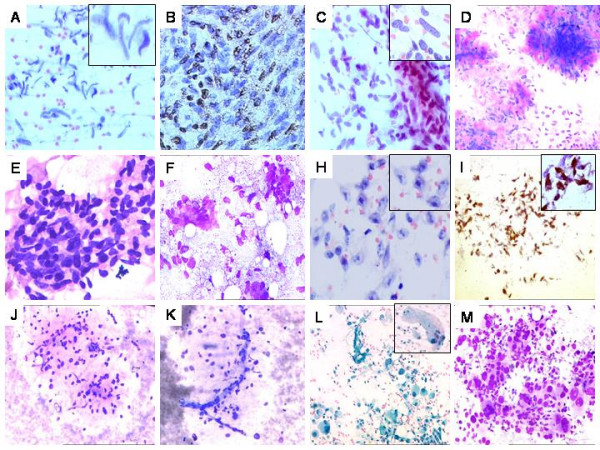
A. Malignant peripheral nerve sheath tumor. Hypercellular smear showing loosely cohesive spindly cells with indented, 'serpentine' nuclei Pap × 400. B. Tumor cells showing S-100 positivity on cellblock preparation. DAB × 400. C. Leiomyosarcoma. Hypercellular smear showing fragments and singly scattered cells with blunt-ended nuclei. Pap × 400. D. Endometrial stromal sarcoma (ascertained on biopsy). Hypercellular smear with ovoid cells and abundant metachromatic basement membrane material. MGG × 200. F. Synovial sarcoma. Hypercellular smear with oval to elongated cells exhibiting overlapping. Pap × 200. G. A recurrent synovial sarcoma showing biphasic cellular pattern and eosinophilic material on smears. MGG × 400. H. Melanoma of soft parts. Cellular smear with spindly cells displaying prominent nucleolisation (inset). Pap × 400. I. Strong S-100 positivity on smears. DAB × 400 (Inset, S-100 highlighting spindly processes. DAB × 1000). J. Myxofibrosarcoma. Moderately cellular smear with oval cells admixed with metachromatic myxoid stroma. MGG × 200. K. Curvilinear vascular pattern with cells around. MGG × 400. L. Pleomorphic Rhabdomyosarcoma. Hypercellular smear with markedly pleomorphic cells. Pap × 400. Inset showing a pleomorphic rhabdomyoblastic cell. Pap × 400. M. Pleomorphic sarcoma not otherwise specified (NOS). Hypercellular smear with markedly pleomorphic cells against a hemorrhagic background. MGG × 400.

**Figure 4:**
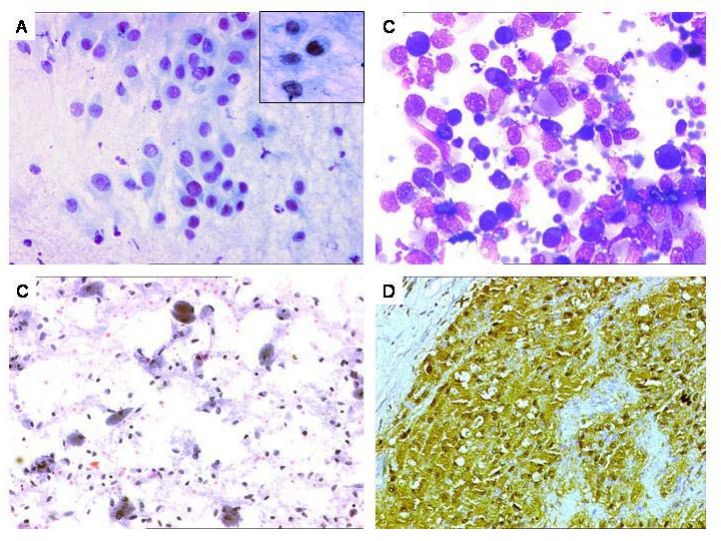
Tumors with epithelioid/polygonal shapes, exactly categorized on biopsy. A. Epithelioid sarcoma (recurrent case). Hypercellular smear with polygonal cells, scattered singly, displaying fine vacuoles in a minority of cells (inset). Pap × 400. B. Anaplastic large cell lymphoma. Hypercellular smear with singly scattered cells revealing moderate to abundant, eosinophilic to finely vacuolated cytoplasm and pleomorphic nuclei. MGG × 400. C. Malignant granular cell tumor. Hypercellular smear with loosely cohesive and singly scattered cells exhibiting ovoid nuclei and abundant, granular, eosinophilic cytoplasm. Pap × 200. C. Diffuse S-100 positivity in tumor cells on IHC on biopsy. DAB × 200.

Out of 7 cases of tumors with epithelioid morphology, all were malignant. The cytological diagnoses included 1 case of an epithelioid sarcoma and 1 case of an epithelioid MPNST. Remaining 5 cases were labeled as sarcomas with an epithelioid morphology. On histopathology, 1 of these turned out as a malignant granular cell tumor; 1 epithelioid leiomyosarcoma; 1 *Alk*1 positive anaplastic large cell lymphoma (ALCL) and another 1 as a pleomorphic sarcoma (NOS). One case turned out to be a myxoid liposarcoma with round/polygonal cells, the latter that were seen in the smears. In the myxoid category, both the tumors were designated as myxofibrosarcomas. (Figures 1, 2, 3, 4). While one of these was in the thigh of a 60 years old male, the other case that turned out as a pleomorphic sarcoma, myxoinflammatory type on biopsy, was identified in the leg of a 52 years old male. (Table No. 1, 2, 3, 4)

**Table 1:** Various cases of spindle cell tumors with FNAC and histopathologic diagnoses

**Sr No.**	**Age**	**Gender**	**Site**	**FNAC Diagnosis**	**Histopathology Diagnosis**	**Concordance**
1	43	M	Thigh	Spindle cell sarcoma	Synovial sarcoma	1
2	30	M	Thigh	Spindle cell sarcoma	Synovial sarcoma	1
3	62	F	Retroperitoneum	Spindle cell sarcoma	NK	3
4	55	F	Chest wall	Spindle cell sarcoma	NK	3
5	65	M	Retroperitoneum	Spindle cell sarcoma	NK	3
6	64	M	Axilla	Spindle cell sarcoma	NK	3
7	56	M	Leg	Spindle cell sarcoma	Spindle cell sarcoma	1
8	57	M	Retroperitoneum	Spindle cell sarcoma	Spindle cell sarcoma	1
9	21	M	Skull	Spindle cell sarcoma	MPNST	1
10	34	F	Iliac Fossa	Spindle cell sarcoma	NK	3
11	29	M	Heel	Synovial sarcoma	Synovial sarcoma	1
12	27	F	Uterus	Leiomyosarcoma	endometrial stromal sarcoma	1
13	56	M	Abdominal wall	Spindle cell tumor	Spindle cell sarcoma	2
14	26	M	Arm	Spindle cell tumor	MPNST	2
15	54	M	Iliac Fossa	Spindle cell tumor	GIST	2
16	48	M	Neck	Leiomyosarcoma	Leiomyosarcoma	1
17	37	M	Pleura	Spindle cell tumor	Schwannoma	2
18	50	M	Posterior Mediastinum	MPNST	MPNST	1
19	62	M	Forearm	Spindle cell tumor-Neurogenic	MPNST	2
20	48	F	Uterus	Spindle cell sarcoma	Leiomyosarcoma	1
21	40	F	Intra abdominal	Spindle cell tumor	NK	3
22	50	F	Uterus	Sarcoma vs poorly differentiated carcinoma	Leiomyosarcoma	1
232	50	F	Uterus	Leiomyosarcoma	Leiomyosarcoma	1
4	34	M	Paraspinal	Melanoma vs MPNST	Pigmented schwannoma	4
25	51	M	Intra abdominal	Spindle cell tumor	DFSP	2
26	25	F	Lumbar	MPNST	MPNST	1
27	25	M	Iliac Fossa	Spindle cell sarcoma	NK	3
28	51	M	Arm	Spindle cell tumor	Proliferative Fascitis	2
29	40	M	Heel	Clear cell sarcoma/Melanoma	Clear cell sarcoma/Melanoma	1
30	52	F	Leg	Synovial sarcoma	Synovial sarcoma	1
31	66	M	Suprascapular	Spindle cell sarcoma	Spindle cell sarcoma	1
32	51	M	Arm	Spindle cell sarcoma	Leiomyosarcoma	1
33	66	M	Neck	Spindle cell tumor	NK	3
34	29	F	Ovary	Spindle cell sarcoma	Spindle cell sarcoma	1
35	22	F	Intra abdominal	Spindle cell lesion	Fibromatosis	2
36	22	M	Maxilla	Spindle cell sarcoma	MPNST	1
37	68	M	Foot	Spindle cell sarcoma	Spindle cell sarcoma	1
38	35	M	Maxilla	Spindle cell lesion	Spindle cell sarcoma	2

**Sr No.**	**Age**	**Gender**	**Site**	**FNAC Diagnosis**	**Histopathology Diagnosis**	**Concordance**

39	35	F	Thigh	Spindle cell sarcoma	Fibrosarcoma	1
40	39	F	Thigh	Spindle cell sarcoma	Spindle cell sarcoma	1
41	35	F	Thigh	Spindle cell sarcoma	Fibromatosis	4
42	28	F	Scapula	Spindle cell sarcoma: Synovial/vs MPNST	Synovial sarcoma	1
43	47	M	Occiput	Spindle cell tumor	Pleomorphic sarcoma	2
44	28	M	Thorax	Spindle cell lesion	Neurofibroma	2
45	20	F	Thigh	Spindle cell sarcoma	MPNST	1
46	40	M	Thigh	Spindle cell sarcoma	Spindle cell sarcoma	1
47	32	F	Neck	Spindle cell sarcoma	Spindle cell sarcoma	1
48	25	F	Arm	Spindle cell sarcoma	Synovial sarcoma	1
49	55	F	Lung	Spindle cell tumor	Spindle cell tumor	1
50	13	M	Leg	Spindle cell sarcoma	MPNST	1
51	30	M	knee	Synovial sarcoma	Synovial sarcoma	1
52	32	M	Neck	Spindle cell sarcoma	Synovial sarcoma	1
53	30	F	Neck	Spindle cell lesion	Spindle cell sarcoma	2
54	24	F	Poplitial Fossa	Spindle cell tumor	Synovial sarcoma	2
55	40	F	Thigh	Spindle cell sarcoma	NK	3
56	56	M	Leg	Clear cell sarcoma/melanoma	Clear cell sarcoma/melanoma	1
57	23	M	Foot	Spindle cell sarcoma	Synovial sarcoma	1
58	67	M	Neck	Spindle cell sarcoma	Spindle cell sarcoma	1
59	47	F	Arm	Spindle cell tumor	Schwannoma	2
60	22	M	Popliteal Fossa	Spindle cell sarcoma	MPNST	1

**Key**: **Concordance**: 1: Concordant; 2: Not specified; 3: Couldn't be ascertained; 4: Discordant M: Male, F: Female

**Table 2:** Various cases of round cell tumors with FNAC and histopathologic diagnoses

**Sr No.**	**Age**	**Gender**	**Site**	**FNAC Diagnosis**	**Histopathology Diagnosis**	**Concordance**
1	2	M	Orbit	Rhabdomyosarcoma	Rhabdomyosarcoma	1
2	14	M	Hand	Rhabdomyosarcoma	Rhabdomyosarcoma	1
3	11	M	Chest wall	Round cell sarcoma	Rhabdomyosarcoma	1
4	18	F	Orbit	Rhabdomyosarcoma	Rhabdomyosarcoma	1
5	2	M	Thigh	PNET/Ewing's sarcoma	PNET/Ewing's sarcoma	1
6	27	M	Thigh	PNET/Ewing's sarcoma	PNET/Ewing's sarcoma	1
7	25	M	Iliac Fossa	PNET/Ewing's sarcoma	PNET/Ewing's sarcoma	1
8	25	M	Iliac Fossa	PNET/Ewing's sarcoma	PNET/Ewing's sarcoma	1
9	30	F	Neck	Sarcoma – Epithelioid	PNET/Ewing's sarcoma	1
10	19	M	Thigh	Malignant round cell tumor	PNET/Ewing's sarcoma	1
11	46	F	Neck	Malignant round cell tumor	PNET/Ewing's sarcoma	1
12	5	F	Thigh	Rhabdomyosarcoma	Rhabdomyosarcoma	1
13	16	M	Maxilla	Rhabdomyosarcoma	Rhabdomyosarcoma	1
14	17	F	Thigh	Rhabdomyosarcoma	Rhabdomyosarcoma	1
15	18	M	Scalp	Rhabdomyosarcoma	Rhabdomyosarcoma	1
16	3	M	Leg	Rhabdomyosarcoma	Rhabdomyosarcoma	1
17	19	M	Neck	Rhabdomyosarcoma	Rhabdomyosarcoma	1
18	1	M	Paraspinal	Round cell sarcoma	Rhabdomyosarcoma	1
19	30	F	Gluteal	PNET/Ewing's sarcoma	PNET/Ewing's sarcoma	1
20	5	F	Scapula	Malignant round cell tumor	Neuroblastoma	1
21	8	M	Leg	Malignant round cell tumor	Rhabdomyosarcoma	1
22	9	M	Intra abdominal	Malignant round cell tumor	Rhabdomyosarcoma	1
23	2	M	Orbit	Malignant round cell tumor	Retinoblastoma	1
24	3	M	Gluteal	Round cell sarcoma	NK	3
25	4	F	Chest wall	PNET/Ewing's sarcoma	PNET/Ewing's sarcoma	1
26	1	F	Orbit	Neuroblastoma	Neuroblastoma	1
27	22	M	Scalp	Malignant round cell tumor	Granulocytic sarcoma	1
28	13	M	Chest wall	PNET/Ewing's sarcoma	PNET/Ewing's sarcoma	1
29	20	F	Axilla	PNET/Ewing's sarcoma	PNET/Ewing's sarcoma	1
30	35	M	Iliac Fossa	PNET/Ewing's sarcoma	PNET/Ewing's sarcoma	1
31	16	M	Iliac Fossa	Malignant round cell tumor	DSRCT	1
32	6	F	Maxilla	Rhabdomyosarcoma (alveolar)	Rhabdomyosarcoma (alveolar)	1

**Key**: **Concordance**: 1: Concordant; 2: Not specified; 3: Couldn't be ascertained; 4: Discordant M: Male, F: Female

**Table 3:** Various cases of epithelioid tumors with FNAC and histopathologic diagnoses

**Sr No.**	**Age**	**Gender**	**Site**	**FNAC Diagnosis**	**Histopathology Diagnosis**	**Concordance**
1	68	M	Back	Epithelioid sarcoma	Epithelioid sarcoma	3
2	47	M	Thigh	Epithelioid MPNST	Epithelioid MPNST	1
3	28	M	Leg	Sarcoma – Epithelioid	Leiomyosarcoma	1
4	58	F	Thigh	Sarcoma – Epithelioid	Myxoid liposarcoma	1
5	65	M	Shoulder	Sarcoma – Epithelioid	Pleomorphic sarcoma (NOS)	1
6	43	M	Thigh	Sarcoma – Epithelioid	Malignant Granular cell tumor	1
7	16	M	Elbow	Sarcoma – Epithelioid	Anaplastic Large cell Lymphoma	1

**Key: Concordance**: 1: Concordant; 2: Not specified; 3: Couldn't be ascertained; 4: Discordant M: Male, F: Female

**Table 4:** Various cases of pleomorphic tumors with FNAC and histopathologic diagnoses

**Sr No.**	**Age**	**Gender**	**Site**	**FNAC Diagnosis**	**Histopathology Diagnosis**	**Concordance**
1	49	M	Scapula	Pleomorphic cell sarcoma	Pleomorphic sarcoma (NOS)	1
2	45	M	Chest wall	Pleomorphic sarcoma, myogenic	Pleomorphic sarcoma rhabdomyosarcoma	1
3	19	M	Leg	Pleomorphic cell sarcoma	Pleomorphic sarcoma (NOS)	1
4	50	F	Chest wall	Pleomorphic Sarcoma vs poorly differentiated carcinoma	NK	3
5	37	F	Thigh	Pleomorphic cell sarcoma	Osteosarcoma	1
6	9	F	Arm	Pleomorphic cell sarcoma	Osteosarcoma	1
7	19	M	Leg	Pleomorphic cell sarcoma; Osteosarcoma	Osteosarcoma	1
8	33	M	Forearm	Pleomorphic rhabdomyosarcoma	Pleomorphic rhabdomyosarcoma	1
9	39	M	Temporal region	Pleomorphic cell sarcoma	Pleomorphic liposarcoma	1
10	55	M	Retroperitoneum	Pleomorphic cell sarcoma	NK	3
11	60	F	Inguinal	Pleomorphic cell sarcoma	Pleomorphic sarcoma (NOS)	1
12	50	M	Chest wall	Pleomorphic cell sarcoma	Pleomorphic sarcoma (NOS)	1

**Key: Concordance**: 1: Concordant; 2: Not specified; 3: Couldn't be ascertained; 4: Discordant M: Male, F: Female

Twenty-eight cases (22%) presented with metastatic deposits, commonly in lymph nodes (16 cases, 57.1%), followed by liver and lungs as the next commonest sites. Highest numbers of cases revealing metastatic deposits were of spindle cell type. However, the highest percentage of cases was of epithelioid category (28.5%), followed by pleomorphic (25.5%), spindle cell (25%), round cell category (18.75%) and finally; lipomatous tumors (14.2%). In terms of exact subtypes, RMS (6 cases) was the commonest tumor to metastasize, followed by leiomyosarcoma (5 cases), synovial sarcoma (4 cases) and pleomorphic sarcoma (4 cases).

## Discussion

Soft tissue tumors (STTs) have been diagnosed with the 'time-honored' histopathology that is recognized as the 'gold standard' for their evaluation. However, in the current era, where 'needle is preceding the scalpel' and the biopsy material is getting limited, it would be prudent to discuss the role and scope of FNAC in diagnosing STTs. The role of FNAC has mostly been noted in soughting recurrent and metastatic STT cases [6-8]. Nevertheless, there are studies indicating its role in primary diagnosis of these tumors [3-5]. The present study was carried out to highlight various aspects of cytological diagnosis of STTs, in a series of 127 cases, wherein maximum cases (50%) were submitted exclusively for a primary diagnosis. An increased number of overall malignant (84.25%) vs. benign cases (12.5%) in our series were comparable to a study by Miralles et al [10] and contrasting to another other study [11]. The reason was the predominance of referral cases, including a greater proportion of sarcomas that our center receives. Moreover, non-neoplastic lesions were excluded in this study.

In terms of diagnosis, complex heterogeneity of STTs is known to be a limiting factor in their exact categorization. With the advent of ancillary techniques like IHC, flow cytometry, cytogenetics and molecular techniques, the objectivity of diagnosing a STT has been considerably enhanced. In a study, Kilpatrick et al [4] have presented the extent to which cytology can be utilized in effective diagnosis of STTs. Subsequently, there have been studies highlighting application of the ancillary techniques in exact sub typing of these tumors [7,8,12,13]. Apart from providing material for these studies, FNAC is easy, safe and cost effective. While the fear of tumor seedling is hardly noted, its effectivity is best exemplified with more access to mass lesions in form of multiple aspirates [4]. In our series, none of the cases had complications or tumor seedlings.

In terms of diagnostic efficacy, 100% sensitivity and 87% specificity in our cases was comparable to results of Nagira et al [11], wherein the respective values were 92% and 97% respectively. In another series, Wakely et al [14] reported 100% sensitivity and 97% specificity in STT diagnosis with FNAC. Layfield et al [3] achieved 95% sensitivity and specificity while dealing with these lesions. In our study, 2 cases (1.57%) were false positive (FP) and none was false negative (FN). While a study on 517 STT aspirates by Akerman et al [15] revealed a 2.9% false positive rate, the subsequent studies by Wakely et al [14] and by Kilpatrick et al [4] yielded a single case of false negativity and nil false positivity. This was in contrast to a study by Nagira et al [11], who identified higher figures for false positivity and false negativity. Our results were comparable to the documented range of < 1%–5% (FP) and 2–15% (FN) [3,4,11,16]. Nil FN in our series was due to the fact that 16 cases were diagnosed as 'unsure'/not specified. Out of these 8 turned out to be malignant, 1 of intermediate malignancy and 5 benign, on biopsy. An overall concordance of 98% is comparable to results of Shah et al [17].

A basic cytological approach towards making a STT diagnosis begins with the familiarity with normal structures, along with myxoid or metachromatic stromal fragments and a variety of dyscohesive cells like spindly, round, pleomorphic, polygonal that are indicators of a STT, on aspirates [9,18]. These features are integrated with clinico radiological findings. The value of radiological findings has been fairly discussed [11,19]. Infact, improved localization of the ST lesion with radiological techniques has revolutionized the amount of aspirate one can achieve, especially in deep-seated lesions. An adequate aspirate is analyzed and non-neoplastic lesions are sorted out. This is followed by placing neoplastic lesions in benign, borderline/intermediate and malignant groups. This approach was followed in the present study, with the exclusion of secondary soft tissue deposits of carcinomas. The sarcomas were not graded similar to a study by Nagira et al [11], even though attempts at cytologic grading of sarcomas have been made [20]. In their study, Mathur et al [20] identified a low concordance of cytological grading in cases of grade 1 vs. high-grade sarcomas. Our series focused upon the value of cytomorphological sub typing into the six categories, as noted earlier [9,11]. While all 32 cases of round cell category; 7 of epithelioid type; 12 of pleomorphic type and 2 of myxoid type were placed into malignant groups, 44 of the total 60 cases (73.3%) of spindle cell type could be labeled as sarcomas. Of the remaining 16 cases, 8 turned out to be malignant; 2 of borderline malignancy and 4 as benign. Remaining 2 cases could not be ascertained, in view of lack of biopsy results. The relationship between cytological categorization with benignancy or malignancy of the subtypes was statistically significant. Among the lipomatous group, more cases (10/14) were benign than malignant (4/14). (p < 0.05). While the results for round cell, pleomorphic and lipomatous tumors were comparable to the findings of Nagira et al [11]; we observed more cases of spindle and all of polygonal and myxoid types, as malignant. The reasons include referral of mostly malignant cases to our centre.

In terms of exact sub typing, which was offered in 58 cases, all lipomatous and 70% pleomorphic tumors were suitably categorized. One case of a pleomorphic lipoma showed presence of mature fibroadipose fragments with 'floret-like' giant cells, displaying nuclear molding. Presence of this tumor in a superficial location i.e. neck, in an elderly male, was a helpful clue in ruling out a liposarcoma [21]. 70% of pleomorphic sarcomas were categorized in congruence with histopathology. Four out of 10 cases could not be categorized, even on histology with IHC. The reason is the referral of poorly differentiated sarcomas to our center. In their study, Berardo et al [22] concluded lack of any cytological features that could differentiate between malignant fibrous histiocytomas with other pleomorphic sarcomas.

Further, 21 cases of round cell tumors, 13 of spindle cell tumors; 2 cases of myxoid type and 2 cases of epithelioid type were ascertained an exact subtype. Maximum cases in the round cell group were of rhabdomyosarcoma and PNET/Ewing's. These diagnoses were confirmed with desmin positivity in the former and CD99 positivity in few of the latter cases. One case of an RMS, with an alveolar component, was formed in view of presence of several 'wreath-like' giant cells. This was confirmed with positive desmin and myogenin staining on ICC, along with positive Myo-D1 expression, on IHC. Further, positive PAX3-FKHR analysis confirmed the alveolar component [23]. One case of soft tissue neuroblastomas showed neuropils and false rosettes. This was objectively confirmed with NSE positivity and MIC2 negativity. Neuroblastomas were lesser in number in our study, as these tumors are well diagnosed with imaging findings, biochemical tests and biopsy, at our Center. FNAC was carried out in few cases.

Among spindle cell tumors, specific diagnoses were assigned in few cases viz. leiomyosarcoma, MPNST, synovial sarcoma in 3 cases and melanoma in 1 case. While the former 2 were substantiated with clinical details, including location, synovial sarcoma was diagnosed based on specific cytomorphological features. Diagnosis of synovial sarcoma in 3 cases included 2 metastatic cases and one recurrent case. Cytomorphology of synovial sarcoma on cytology is complex. In unusual situations, ICC and t (x: 18) translocations (SYT-SSX) can be useful in forming this exact subtype [13,24]. One case of melanoma of soft parts was ascertained with prominently nucleolated spindly cells and few polygonal cells that exhibited diffuse S-100 positivity. This was confirmed with additional HMB45 positivity, on IHC [25]. On biopsy, level of exact subyping was significantly increased in spindle cell tumors. Another case of an endometrial stromal sarcoma was presumed to be a leiomyosarcoma, on FNAC. Retrospectively, presence of hypercellular smears with ovoid cells and prominent basement membrane-like material reminiscent of proliferating vessels were identified as useful features. Recently, a similar case has been reported [26]. Out of 16 cases that were not classified, 4 turned out benign on biopsy. The remaining 8 cases included 2 of intermediate malignancy, namely, 1 case, each of a dermatofibrosarcoma protuberans (DFSP) and fibromatosis. Another 1 case was of proliferative fasciits, on biopsy. In case of adequate material and typical clinical settings, diagnosis of a DFSP can be suggested [27]. The other daunting tumor is fibromatosis that is recognized as a pitfall in STT diagnosis [3,5,13]. This was identified as 1 false positive case in our study, along with another case of MPNST that turned out to be a pigmented schwannoma, on histopathology.

While 2 myxoid tumors were termed as myxofibrosarcomas, one of these turned out to be a pleomorphic sarcoma, myxoinflammatory type. Lack of exact categorization of sarcomas with epithelioid morphology (5 cases) was due to limited cellularity and tumor heterogeneity. One case labeled as sarcoma with epithelioid morphology turned out to be a myxoid liposarcoma, with a round/polygonal cell component. The remaining 2 cases in this category were unusual. While one case turned out to be a malignant granular cell tumor, the other was an Alk-1 positive ALCL. Granular cell tumors have been recognized as a pitfall in FNAC of breast lesions [28]. The diagnosis was ascertained with S100 positivity, on biopsy [29]. The case of an *Alk1 *+ ALCL was noted in a young adolescent male, with multifocal lesions Smears showed dyscohesive polygonal cells with multilobated nuclei and moderate amount of finely vacuolated cytoplasm [30]. Positivity for LCA, CD30, EMA and *Alk-1 *confirmed this diagnosis, on biopsy. The morphological overlapping was observed between epithelioid and round cell type; between spindle cell and polygonal type in case of melanoma of soft parts, as well as between pleomorphic and lipomatous group in 2 pleomorphic liposarcomas. Nonetheless, cytomorphological categorization helped in building up an algorithmic approach towards exact sub typing.

While Costa et al [31] achieved 20.9% rates in terms of exact sub typing of sarcomas, we, like Nagira et al [11], observed an overall higher rate. In their review, Kilpatrick et al [4] have presented a range of 21–74% of exact sub typing of sarcomas, based on various studies. Exact sub typing can be increased with application of ICC [13]. In the present study, routine ICC was attempted in limited cases; mostly of the round cell type, as exact sub typing in these cases has a definite bearing on management. Maximum cases that lacked exact sub typing were of the spindle cell type. Thirteen cases were diagnosed as spindle cell sarcomas, even on histology. The reasons in many of these included referral cases with lack of paraffin blocks and negativity of a specific lineage, even with IHC, in the remaining few. Exact sub typing of spindle cell tumors is a limiting factor, even in other studies [9]. Prognostication of soft tissue sarcomas has been recommended with histological grading and staging [32]. Recent studies [33,34] on cytomorphology of leiomyosarcomas and neurogenic tumors constitute an 'add-on' to refining the existing cytomorphology of these subtypes.

Apart from its primary diagnosis; the role of FNAC is recognized in metastatic STT evaluation [7,8]. Evaluation of 'foreign cells' in metastatic locations, in cases with known clinical context, is less challenging than in situations initial metastatic presentations. In such cases, clinical details are imperative before ascertaining a diagnosis. In our series, out of 28 cases with metastatic lesions, maximum metastatic deposits were reported in lymph nodes (55.1%), 'more-so' of the groin, followed by liver and lungs. While lymph nodes have been observed as the commonest metastatic location in earlier studies [7,8], in our study, groin nodes, were found to be more commonly involved. This might be as a result of more STS occurring in extremities. While maximum metastatic cases were of spindle cell type (considering their higher absolute number), the percentage was highest with epithelioid tumors. This is because of tendency of sarcomas, with epithelioid morphology, to have increased chances for metastasis, like carcinomas. An earlier series observed RMS as the commonest metastasizing tumor [8]. In terms of exact subtypes, RMS was the commonly metastasizing tumor in our series.

**To sum up**, FNAC has a definite role in the diagnosis of STTs in primary, recurrent and metastatic lesions, for a timely management. Cytological categorization is effective, especially for sorting out round cell and pleomorphic tumors. Spindle and epithelioid cell tumors are challenging, especially in terms of exact sub typing. Exact sub tying can be enhanced with applications of ICC. Recognition of 'pitfall' lesions like aggressive fibromatosis is vital. Further scope of STT evaluation on cytology can be increased with more studies dealing with application of ancillary techniques on aspirate samples.

## Competing interests

The author(s) declare that they have no competing interests.

## Authors' contributions

**ACK: **Description of data and statistical analysis.

**BDG: **Retrieval of cases, involved in review of cases.

**BR: **Study design, review of cases, data analysis and preparation of the manuscript

**RFC: **Overall supervision and has given the final approval of the manuscript.

## References

[B1] Hospital Based Cancer Registry programme (2001). Tata Memorial Hospital, Mumbai, India.

[B2] Chang AE, Rosenberg SA, Glastein EJ, Antman KH, Vita VD, Hellman S, Rosenberg SA (1989). Sarcomas of soft tissues. Cancer: Principles and Practice of Oncology.

[B3] Layfield LJ, Anders KH, Glasgow BJ, Mira JM (1986). Fine needle aspiration of primary soft-tissue tumors. Arch Pathol Lab Med.

[B4] Kilpatrick SE, Cappellari JO, Bos GD, Gold SH, Ward WG (2001). Is fine-needle aspiration biopsy a practical alternative to open biopsy for the primary diagnosis of sarcoma?. Am J Clin Pathol.

[B5] Dey P, Mallik MK, Gupta SK, Vasishta RK (2004). Role of fine needle aspiration cytology in the diagnosis of soft tissue tumors and tumor like lesions. Cytopathol.

[B6] Trovick CS, Bauer HC, Brosjo O, Skoog L, Soderlund V (1998). Fine needle aspiration (FNA) cytology to diagnose suspected local recurrences of soft tissue sarcoma. Cytopathology.

[B7] Loya AC, Prayaga AK, Arora A, Sundaram C, Rao IS, Uppin SG, Raju GS, Surath A, Rajappa RS (2007). Lymph node metastasis of soft tissue tumors: cytomorphologic study. Acta Cytol.

[B8] Khirwadkar N, Dey P, Das A, Gupta SK (2000). Fine-needle aspiration biopsy of metastatic soft-tissue sarcomas to lymph nodes. Diagn Cytopathol.

[B9] Kilpatrick SE, Geisinger KR (1998). Soft tissue sarcomas: the utility and limitation of fine needle aspiration biopsy. Am J Clin Pathol.

[B10] Miralles TG, Gosalbez F, Menéndez P, Astudillo A, Torre C, Buesa J (1986). Fine needle aspiration cytology of soft-tissue lesions. Acta Cytol.

[B11] Nagira K, Yamamoto T, Akisue T, Marui T, Hitora T, Nakatani T, Kurosaka M, Ohbayashi C (2002). Reliability of fine-needle aspiration biopsy in the initial diagnosis of soft-tissue lesions. Diagn Cytopathol.

[B12] Sapi Z, Antal I, Papi Z, Szendröi M, Mayer A, Jakab K, Pajor L, Bodó M (2002). Diagnosis of soft tissue tumors by fine needle aspiration cytology combined with ancillary techniques. Diagn Cytopathol.

[B13] Rekhi B, Bhatnagar D, Bhatnagar A, Saxena S (2005). Cytomorphological study of soft tissue neoplasms-role of fluorescent immuocytochemistry in diagnosis. Cytopathology.

[B14] Wakely PE, Kneisl JS (2000). Soft tissue aspiration cytopathology. Cancer.

[B15] Akerman M, Rydholm A, Persson BM (1985). Aspiration cytology of soft-tissue tumors. The 10-year experience at an orthopedic oncology center. Acta Orthop Scand.

[B16] Bommer KK, Ramzy I, Mody D (1997). Fine-needle aspiration biopsy in the diagnosis and management of bone lesions: a study of 450 cases. Cancer.

[B17] Shah MS, Garg V, Kapoor SK, Dhaon BK, Gondal R (2003). Fine-needle aspiration cytology, frozen section, and open biopsy: relative significance in diagnosis of musculoskeletal tumors. J Surg Orthop Adv.

[B18] Layfield LJ, Layfield LJ (2002). Cytologic appearance of normal mesenchymal tissues. Cytopathology of bone and soft tissue tumors.

[B19] Einarsdóttir H, Söderlund V, Skoog L, Bauer HC (2003). Dynamic MRI and fine needle aspiration cytology in the evaluation of soft tissue lesions. Skeletal Radiol.

[B20] Mathur SR, Kapila K, Verma K (2003). Accuracy of cytological grading of spindle-cell sarcomas. Diagn Cytopathol.

[B21] López-Ríos F, Alberti N, Pérez-Barrio A, de Agustín PP (2001). Fine-needle aspiration of pleomorphic lipoma. Diagn Cytopathol.

[B22] Berardo MD, Powers CN, Wakely PE, Almeida MO, Frable WJ (1997). Fine-needle aspiration cytopathology of malignant fibrous histiocytoma. Cancer.

[B23] Sorensen PH, Lynch JC, Qualman SJ, Tirabosco R, Lim JF, Maurer HM, Bridge JA, Crist WM, Triche TJ, Barr FG (2002). PAX3-FKHR and PAX7-FKHR gene fusions are prognostic indicators in alveolar rhabdomyosarcoma: a report from the children's oncology group. J Clin Oncol.

[B24] Akerman M, Domanski HA (2007). The complex cytological features of synovial sarcoma in fine needle aspirates, an analysis of four illustrative cases. Cytopathology.

[B25] Paari Murugan, Debdatta Basu, Surendra Kumar, Sadasivan Jagadish (2007). Clear cell sarcoma of the soft parts in the rectus abdominis in child- aspiration cytology of a rare case. Cyto Journal.

[B26] Awasthi A, Rajwanshi A, Sarla M (2007). Fine needle aspiration cytology of low-grade endometrial stromal sarcoma: a case report. Acta Cytol.

[B27] Rekhi B, Burra U, Saxena S (2004). Fine needle aspiration cytology of Dermatofibrosarcoma protuberans – A Case Report. J Cytol.

[B28] Pieterse AS, Mahar A, Orell S (2004). Granular cell tumour: a pitfall in FNA cytology of breast lesions. Pathology.

[B29] Geisinger KR, Kawamoto EH, Marshall RB, Ahl ET, Cooper MR (1985). Aspiration and exfoliative cytology, including ultrastructure, of a malignant granular-cell tumor. Acta Cytol.

[B30] Liu K, Dodd LG, Osborne BM, Martinez S, Olatdioye BA, Madden JF (1999). Diagnosis of anaplastic large-cell lymphoma, including multifocal osseous KI-1 lymphoma, by fine-needle aspiration biopsy. Diagn Cytopathol.

[B31] Costa MJ, Campman SC, Davis RL, Howell LP (1996). Fine-needle aspiration cytology of sarcoma: retrospective review of diagnostic utility and specificity. Diagn Cytopathol.

[B32] (1999). Association of Directors of Anatomic and Surgical Pathology. Recommendations for the reporting of soft tissue sarcomas. Am J Clin Pathol.

[B33] Domanski HA, Akerman M, Rissler P, Gustafson P (2006). Fine-needle aspiration of soft tissue leiomyosarcoma: an analysis of the most common cytologic findings and the value of ancillary techniques. Diagn Cytopathol.

[B34] Klijanienko J, Caillaud JM, Lagacé R (2006). Cytohistologic correlations in schwannomas (neurilemmomas), including "ancient," cellular, and epithelioid variants. Diagn Cytopathol.

